# The association of social support with functional status and physical activity in patients with coronary heart disease from The Heart and Soul study

**DOI:** 10.1186/s12872-025-05338-8

**Published:** 2025-12-30

**Authors:** Abdelaziz Elnaggar, Thomas J. Hoffmann, Mary A. Whooley, Rochelle Szuba, Linda G. Park

**Affiliations:** 1 Department of Community Health Systems, School of Nursing, University of California San Francisco, San Francisco, CA USA; 2 Institute for Human Genetics, University of California San Francisco, San Francisco, CA USA; 3 Departments of Medicine and Epidemiology and Biostatistics, University of California San Francisco, San Francisco, CA USA

**Keywords:** Coronary heart disease, Older adults, Social support, Physical activity, Activities of daily living, Instrumental activities of daily living, Depression, Functional status

## Abstract

**Introduction:**

Coronary heart disease (CHD) is a significant public health problem among older adults that is associated with reduced physical activity (PA) and impaired functional capacity, commonly measured by activities of daily living (ADL) and instrumental activities of daily living (iADL). Maintaining independence in ADLs is critical for older adults' quality of life and healthcare outcomes. While social support is known to improve health outcomes, its long-term impact on PA and functional status in CHD patients has not been fully explored.

**Objective:**

This study aimed to investigate the association between social support and functional outcomes, including PA, in a cohort of long-term survivors with established CHD.

**Methods:**

This study leverages 2 decades of the Heart and Soul Study, a multicenter investigation into mental health factors and clinical outcomes in individuals with CHD. We report a longitudinal analysis of baseline (2000–2002) and 20-year follow-up data (2022) to detect differences in social support and its association with functional status and PA over 20 years of surviving participants. Social support was measured using the 12-item Interpersonal Support Evaluation List (ISEL) subscales. Functional status was assessed using a combination of Katz and Lawton ADL scales, and PA was measured with self-reported data. Logistic regression models assessed associations between social support, ADL, and PA, accounting for change over time (baseline and 20 years), adjusting for sociodemographic factors and depressive symptoms.

**Results:**

The sample included 129 participants (mean age 80.9 years, SD ± 9.1), 72.9% of whom were male. In unadjusted analyses, better appraisal and belonging social support scores were associated with greater independence in iADL. These associations were insignificant after adjusting for sociodemographic characteristics (age, sex, income, race/ethnicity) and depression. Better appraisal and belonging scores were also significantly associated with higher PA. These associations remained significant after adjusting for sociodemographic factors, but did not remain significant after controlling for depression. After adjusting for the same covariates, we found no significant interaction between social support and time (20 years).

**Conclusion:**

This study provides a 20-year perspective on the role of social support in maintaining physical activity and independence in older adults with CHD. Our findings suggest that while social support is linked to better functional status and PA, these benefits are not sustained, accounting for sociodemographic characteristics and depression. Highlighting the need for integrating mental health support alongside social support strategies in long-term interventions to improve functional outcomes.

## Background

Coronary heart disease (CHD) remains a significant public health concern, particularly among older adults [1]. The impact of CHD extends beyond cardiovascular complications, often leading to decreased physical activity (PA) [2] and impaired functional capacity. The latter is frequently measured by activities of daily living (ADL), which are classified into basic ADL (e.g., bathing, dressing, and eating) and instrumental activities of daily living (iADL), such as managing finances and performing housework [3]. Maintaining independent ADLs strongly predicts older adults’ ability to live independently, quality of life (QOL), and healthcare outcomes [3]. In patients with CHD, functional decline can be accelerated due to the cumulative effects of the disease, which contribute to both physical limitations and psychosocial stressors, including depression and reduced social interaction [4]. As ADL performance deteriorates, patients may experience increased reliance on caregivers and healthcare services.

While extensive research has been conducted on CHD and its consequences, the long-term influence of social support on the outcomes of PA and functional status in older adults with CHD remains inadequately explored. Social support has been linked to improved health outcomes across various chronic conditions [5]. Previous studies have demonstrated the potential of social support in mitigating the adverse effects of chronic diseases and enhancing cardiovascular reactivity and function [6]. However, little is known about whether social support continues to shape functional independence and physical activity after two decades of CHD survivorship. The specific impact of these social support elements on ADL and PA in older adults with CHD over an extended period [7] is an opportunity to understand modifiable predictors of important outcomes and behaviors that affect health.

This study leverages 20 years of longitudinal data from the Heart and Soul Study survivors, a robust prospective cohort study of older adults with CHD. The primary objective of this study was to assess the longitudinal association between overall social support and changes in ADL and PA over 20 years in this population. Specifically, we evaluated the individual social support dimensions of appraisal, belonging, and tangible support and their relationship to functional status and PA outcomes.

## Methods

### Study design and setting

This study evaluated surviving participants from the Heart and Soul, a 20-year, multicenter prospective cohort study designed to examine the relationship between psychosocial factors and clinical outcomes in individuals with CHD. Details of the study have been previously published [4, 8–10]. Participants were recruited from two Veterans Affairs Medical Centers, one university medical center, and nine public health clinics in the Community Health Network of San Francisco. Institutional review boards at each site approved the protocol, and all participants provided written informed consent. The Heart and Soul study enrolled 1,024 ambulatory men and women aged 37 to 94 with established CHD between September 2000 and December 2002. Participants were eligible if they had a history of myocardial infarction, angiographic evidence of stenosis of 50% or greater in one or more coronary vessels, evidence of exercise-induced ischemia, or a history of coronary revascularization. Exclusion criteria included having a myocardial infarction in the past six months, inability to walk one block, or planning to move out of the area within three years. The study protocol was approved by the Committee on Human Research at the University of California, San Francisco; the Research and Development Committee at the San Francisco Veterans Affairs Medical Center; the Medical Human Subjects Committee at Stanford University; the Human Subjects Committee at the Veterans Affairs Palo Alto Health Care System; and the Data Governance Board of the Community Health Network of San Francisco. All participants provided written informed consent.

### Recruitment

Of the 1,024 participants enrolled initially at baseline of the Heart and Soul Study, 787 (77%) had died as of February 2022. Of the remaining survivors, 42 had not reconsented for further contact, and 30 were lost to follow-up, leaving 168 (70% of the surviving participants) who remained active in the study. Among these, 129 completed the 20-year follow-up survey by mail. Inclusion in the current study required participants to have data points at baseline (2000–02) and 20-year (2022) follow-up visits. Interested participants were contacted by phone to complete a cognitive assessment using the Pfeiffer Short Portable Mental Status Questionnaire (SPMSQ) [11] as another criterion for inclusion. The SPMSQ is a 10-item questionnaire scored from 0 (best cognitive function) to 10 (worst cognitive function). Participants needed to score ≤ 2 to be deemed cognitively fit [12]. Verbal consent was obtained during the call. At baseline (2000–2002), comprehensive clinical assessments were performed, including comorbidities, biomarkers, and cardiovascular risk factors. At the 20-year follow-up (2022), only psychosocial and functional measures were collected (social support, depression, PA, ADL/iADL), with no repeat clinical or biomarker assessments.

### Data collection

At the baseline and 20-year follow-up exams, participants completed self-report measures of depressive symptoms, social support, functional status (activities of daily living and instrumental activities of daily living), social network, and physical activity. Sociodemographic variables were measured with standardized questionnaires from the baseline data.

Depressive symptoms were measured using the eight-item Patient Health Questionnaire (PHQ-8), excluding the question on suicidal ideation. The PHQ-8 has shown reliability and validity in multiple studies [10], with a Cronbach’s α of 0.82 indicating internal validity and items reliably measure the same concept [13].

Social support was measured using the 12-item Interpersonal Support Evaluation List (ISEL) [14]; higher scores reflect greater perceived social support. ISEL includes three subscales: tangible, appraisal, and belonging. Tangible support measures the perceived availability of material aid (e.g., having someone to help with tasks); appraisal support measures the perceived availability of someone to talk to about personal problems; and belonging support measures the perceived availability of people to do things with (e.g., social activities). Each subscale consists of four question items. The original ISEL questionnaire contains 40 items, with recognized internal consistency of Cronbach's α coefficients ranging from 0.70 to 0.80 across its subscales [15]. Additionally, the test–retest reliability, with intraclass correlation coefficients ranging from 0.63 to 0.85 [16], shows stable and consistent results over time. The shorter 12-item version of the ISEL maintains the integrity and reliability of the original 40-item version [17–19].

ADLs were assessed using a questionnaire combining elements from the Katz Index of Independence in Activities of Daily Living (Katz ADL) [20] and the Lawton Instrumental Activities of Daily Living (Lawton iADL) [21]. The Katz scale includes six items on essential activities, including bathing, dressing, toileting, mobility, continence, and feeding, with scores ranging from 0 (dependent) to 6 (independent). The Lawton scale assesses more complex activities, including using the telephone and handling finances, with scores ranging from 0(dependent) to 8 (independent). We dichotomized this scale so that a score of 1 represented independence (≤ 2.5) and 0 represented dependence (> 2.5) [22, 23]. The reliability of both scales has been rigorously tested, showing excellent reproducibility [24, 25].

Physical activity was measured using a self-reported question: "Which of the following statements best describes how physically active you have been during the last month?" Responses ranged from 'not at all active' to 'extremely active' (≥ 5 times per week). Participants were deemed physically inactive if they worked out ≤ 4 times monthly. This classification aligns with trends observed in the Heart and Soul cohort and has been shown to predict CHD outcomes [26]. The single-item self-reported PA measure has shown strong construct validity and reliability. Prior studies demonstrated correlations with treadmill-based cardiorespiratory fitness, health risk profiles [27], and both questionnaire-based assessments (e.g., GPAQ) and functional performance outcomes [28, 29], supporting its utility as a robust tool for assessing PA in large cohorts.

### Statistical analysis

Categorical variables are presented as counts and proportions. If skewed, continuous variables are reported as means (and standard deviations) or medians (and interquartile ranges).

To test for associations between social support variables and outcomes (ADL and PA) over time (baseline and 20 years), we used logistic mixed effects models using the R v4.4.0 [30] package lme4 v1.1.35.5 [31]. We dichotomized our outcomes by ADL ≤ 2 and PA ≥ 2, i.e., so that more independent or more physically active took the value of 1 (vs. 0). Model 1 included effects for each of the ISEL scores in turn, an effect for time at 20 years, a random effect for individual, and (a) no additional covariates, (b) sociodemographic covariates for age, sex, income, and race/ethnicity, and (c) including all (b) plus the PHQ-8 depression scale. To see if there was any association difference over time, Model 2 included all terms from Model 1 plus the interaction between the ISEL score term and the outcome, which differed between baseline and the 20-year follow-up. Results were reported as odds ratios (OR) with 95% confidence intervals (CI). Statistical significance was set at *p* < 0.05.

## Results

One hundred twenty-nine participants were included in the analysis with complete baseline (2000–2002) and 20-year follow-up (2022) data. The mean age of the participants was 80.9 ± 9.1 years, and 72.9% (*n* = 94) were male. In this study, 64.3% (n = 83) have intact cognition with SPMSQ score < 2. The cohort's baseline characteristics are summarized in Table 1.

**Table 1:** Participants Characteristics at 20-year follow-up

Participants Characteristics (*N* = 129)	
	**N (%), or mean ± SD**
Age (y), mean/SD	80.0 ± 9.1
≥ 80 (y)	71 (55.0)
Male gender	94 (72.9)
Race	
White	73 (56.6)
African American	18 (14.0)
Asian or Pacific Islander	21 (16.3)
Hispanic, Latino	12 (9.3)
Other	5 (3.9)
Socioeconomic Status (baseline)	
Income < $ 20 K	70 (54.3)
Education ≤ high school	71 (55.0)
Depressive Symptoms (PHQ-8)	
None	73 (56.6)
Mild	30 (23.3)
Moderate	12 (9.3)
Severe	9 (7.0)
Physically active	62 (48.1)
Independent functional status	
Activities of Daily Living	107 (82.9)
Instrumental Activities of Daily Living	84 (65.1)

### Social support and iADL

**Fig. 1:**
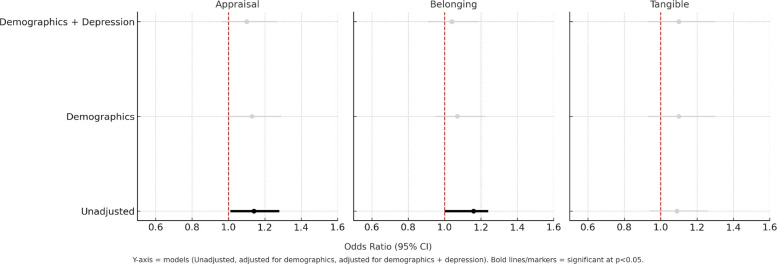
Association between Social Support Elements and iADL (Model 1)

**Fig. 2:**
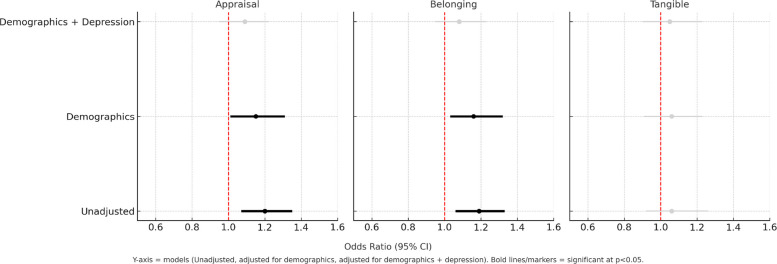
Association between Social Support Elements and Physical Activity (Model 1)

In Model 1, which examines the overall effect of social support, better appraisal and belonging subscales scores were significantly associated with better iADL (Lawton) scores, with an OR of 1.14 and 1.16, respectively (*p* < 0.05), when unadjusted. However, when adjusting for sociodemographic covariates (age, sex, income, race/ethnicity), the association was no longer statistically significant (*p* > 0.05, Table 2, Fig. 1). In Model 2, which included interaction terms to examine whether the association between each social support term and iADL changed over time, a significant interaction (OR = 0.65, *p* < 0.05) was observed for tangible when unadjusted. This interaction did not remain statistically significant after adjusting for additional covariates (*p* ≥ 0.05, Fig. 2).

**Table 2:** Association between social support elements and iADL (Lawton)

Unadjusted for additional covariates				
Covariate	Model 1**		Model 2***	
	OR_main effect_	95% CI	OR_interaction_	95% CI
Appraisal	**1.14**	(1.01, 1.28)*	0.94	(0.73, 1.22)
Belonging	**1.16**	(1.00, 1.24)*	0.84	(0.63, 1.13)
Tangible	1.09	(0.94, 1.26)	**0.65**	(0.46, 0.92)*
**After adjustment for age, sex, income, race/ethnicity**				
Appraisal	1.13	(0.99, 1.29)	1.07	(0.78, 1.46)
Belonging	1.07	(0.95, 1.22)	0.91	(0.65, 1.28)
Tangible	1.10	(0.93, 1.30)	0.75	(0.50, 1.11)
**After additionally adjusting for depression**				
Appraisal	1.10	(0.96, 1.27)	1.06	(0.78, 1.46)
Belonging	1.04	(0.91, 1.19)	0.92	(0.66, 1.29)
Tangible	1.10	(0.93, 1.30)	0.78	(0.53, 1.16)

^*^p <.05

^**^Model 1: The overall effect of each social support subscale on outcomes across the whole period

^***^Model 2: Interaction terms to examine whether the effect of social support on outcomes differs between baseline and at 20 years 20-year follow-up

### Social support and physical activity

**Fig. 3:**
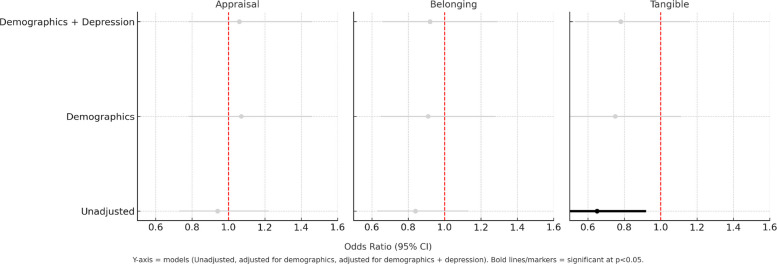
Association between Social Support Elements and iADL (Model 2)

**Fig. 4:**
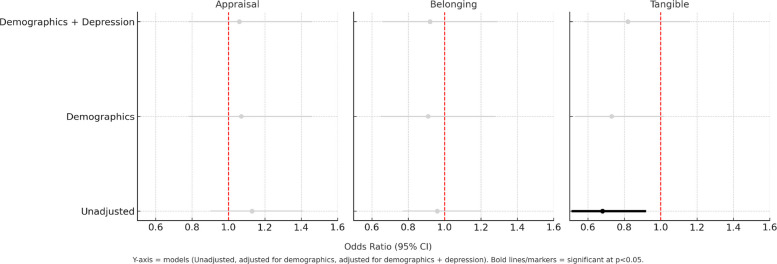
Association between Social Support Elements and Physical Activity (Model 2)

Similarly, in Model 1, better appraisal and belonging scores showed significant associations with higher PA scores, with ORs of 1.20 and 1.19, respectively (*p* < 0.05), when unadjusted for covariates. These associations remained significant after adjusting for age, sex, income, and race/ethnicity. However, this significance was lost after adjusting for depression (Table 3, Fig. 3). When testing for an interaction in Model 2, only the tangible score was significant when unadjusted; nonetheless, this association was lost after adjusting for additional covariates (Fig. 4).

**Table 3:** Association between social support elements and physical activity

Unadjusted for additional covariates				
Covariate	Model 1**		Model 2***	
	OR_main effect_	95% CI	OR_interaction_	95% CI
Appraisal	**1.20**	(1.07, 1.35)*	1.13	(0.90, 1.41)
Belonging	**1.19**	(1.06, 1.33)*	0.96	(0.77, 1.20)
Tangible	1.06	(0.92, 1.26)	**0.68**	(0.51, 0.92)*
**After adjustment for age, sex, income, race/ethnicity**				
Appraisal	**1.15**	(1.01, 1.31)*	1.16	(0.89, 1.50)
Belonging	**1.16**	(1.03, 1.32)*	1.01	(0.78, 1.30)
Tangible	1.06	(0.91, 1.23)	0.73	(0.53, 1.02)
**After additionally adjusting for depression**				
Appraisal	1.09	(0.95, 1.22)	1.13	(0.86, 1.49)
Belonging	1.08	(0.95, 1.23)	1.00	(0.77, 1.29)
Tangible	1.05	(0.90, 1.23)	0.82	(0.58, 1.16)

^*^ *p* <.05

^**^Model 1: The overall effect of each social support subscale on outcomes across the whole period

^***^Model 2: Interaction terms to examine whether the effect of social support on outcomes differs between baseline and at 20 years 20-year follow-up

### Katz score analysis

Initial consideration was given to including the Katz ADL score in the model to evaluate its association with social support. However, the limited cell counts precluded testing for association. At baseline, all participants demonstrated a Katz score above 0, indicating no ADL impairment.

## Discussion

Our study provides longitudinal 20-year insights into the relationship between social support, independent living, and PA among older adults with longstanding CHD. We observed that individuals with higher levels of social support, particularly in appraisal and belonging, were more likely to remain physically active and independent. However, these associations did not hold after accounting for socioeconomic status (SES), age, and depressive symptoms. This suggests that the benefits of social support may be influenced by co-occurring depression. Although social support and depressive symptoms are often described as having a bidirectional relationship, our findings cannot establish causality. In prior analyses from this cohort [10, 32, 33], depressive symptoms have variably appeared as a confounder, mediator, or effect modifier depending on the outcome and analytic framework. In the present study, depressive symptoms may plausibly be a confounder or mediator of the association between social support and functional outcomes. Future longitudinal and mechanistic studies are needed to clarify these complex interrelationships. This also highlights the need for a comprehensive care approach that addresses this population's mental and social factors.

This study found that adjusting for depressive symptoms reduced the association between social support and both PA and iADLs. Indicating that depressive symptoms could explain much of the observed relationship. Tangible support appeared more relevant at later follow-up in unadjusted models, but this effect did not remain significant after accounting for sociodemographic factors and depression. This finding differs from previous research showing that social support has long been associated with improved health outcomes by enhancing the overall well-being of adults with chronic conditions [34–36]. However, our findings align with other studies that a lack of social support can lead to a cycle of depressive symptoms, lower PA, and increased dependency on others for ADLs [37–40].

The clinical implications of this study reinforce the need for a comprehensive care approach in managing older adults with CHD that addresses SES together with social support and mental health, particularly depression. These interventions can address the higher incidence of mental illness [41–43] within this population by integrating screenings and support services within cardiac rehabilitation programs and routine visits to general practitioners. Referral to community-based initiatives could foster social connections and psychosocial well-being (e.g., SilverSneakers, YMCA, local gyms).

Our study has several limitations that should be acknowledged. The relatively small sample size and the homogeneity of the cohort, primarily older adults who survived CHD for over two decades, may limit the generalizability of the findings and may have impacted our ability to detect associations. The lack of diversity in the sample, particularly in terms of SES and race, might restrict the broader applicability of the results to different populations. Additionally, the reliance on self-reported measures for social support and PA over 20 years, although significant interactions with time were not observed. Although follow-up assessments were conducted, social support was measured only at baseline, 5, and 20 years. The 5-year data had substantial missingness with too few complete cases for analysis, so we restricted our analyses to baseline and 20-year data. Lastly, because our sample consists of individuals with CHD who survived two decades, these participants may represent a healthier subgroup, introducing a survival bias.

Further longitudinal studies with larger and more diverse sample sizes are warranted to confirm these findings. Specifically, how different dimensions of social support interact with mental health over time, and how depression may moderate or mediate the impact of social support on PA and functional status. Additionally, a broader range of demographic variables and diverse socioeconomic and racial groups would confirm the external validity of the results. Such research could provide valuable insights into developing tailored interventions that address mental health and social support.

## Conclusion

Our study provides a 20-year perspective on the role of social support in maintaining PA and independent living in older adults with CHD. While initial findings suggested that social support was associated with greater independence and higher PA, this association was no longer significant after adjusting for sociodemographic factors and depression. This indicates that the positive effects of social support may be substantially diminished in the context of depression. Thus, it emphasizes the need for comprehensive care approaches that integrate mental health and social support interventions. Future research should further investigate the interaction between these factors across more diverse populations.

## Data Availability

The dataset analyzed during the current study is not publicly available due to participants’ confidentiality and institutional policies.

## Abbreviations

ADL: Activities of Daily Living
CHD: Coronary Heart Disease
CI: Confidence Interval
iADL: Instrumental Activities of Daily Living
ISEL: Interpersonal Support Evaluation List
OR: Odds Ratio
PA: Physical Activity
PHQ-8: Patient Health Questionnaire-8
QOL: Quality of Life
SES: Socioeconomic Status
SPMSQ: Short Portable Mental Status Questionnaire
VA: Veterans Affairs
